# Screening for novel factors involved in mouse early embryonic development using inhibitor libraries

**DOI:** 10.3389/fcell.2025.1643551

**Published:** 2025-10-17

**Authors:** Hirofumi Nishizono, Masaki Kato

**Affiliations:** ^1^ Research Support Center, Medical Research Institute, Kanazawa Medical University, Uchinada, Japan; ^2^ Data Knowledge Organization Unit, RIKEN Information R&D and Strategy Headquarters, RIKEN, Wako, Japan

**Keywords:** early embryonic development, inhibitor library screening, mouse embryo, embryonic regulatory factors, cathepsin D, CXCR2

## Abstract

Mammalian early embryonic development is regulated by numerous factors, yet not all have been identified. Although omics approaches such as next-generation sequencing and proteomics provide powerful tools, screening methods using inhibitor libraries remain highly effective for identifying novel factors involved in embryonic development. To this end, we developed a novel screening system that combines ultra-superovulation technology with one-cell stage embryos cryopreservation in mice. Using this system, we screened 95 inhibitors to identify factors essential for the development of mouse fertilized eggs and identified 16 factors, including 5 previously known ones. Among the known factors, two were ATPases, and our data confirmed that inhibition of different ATPase types arrested embryonic development at distinct stages. In addition, we discovered novel regulators affecting various developmental stages, including a p53 activator (PRIMA-1), cathepsin D, CXCR2, and potassium channels (SK2 and SK3). Genome editing experiments involving knockout of the cathepsin D and CXCR2 genes further verified the arrest of embryonic development. These results demonstrate that our developed screening method can effectively identify novel factors involved in embryonic development. Application of this approach to additional inhibitor libraries and other species may facilitate the discovery of further species-specific regulators of early embryonic development.

## 1 Introduction

Infertility affects approximately one in six individuals at some point in their lives, posing a significant challenge in both developed and developing countries (Cox et al., 2022). Advancing assisted reproductive technologies requires a comprehensive understanding of critical processes such as fertilization, early embryonic development, and implantation—particularly through the identification of novel regulatory factors and elucidation of their mechanisms of action (Sang et al., 2023). In particular, discovering factors involved in early embryonic development is essential for improving *in vitro* fertilization (IVF) and embryo culture techniques.

Recent advances in proteomics and genomics have led to the identification of many novel factors involved in embryonic development (Deutsch et al., 2014; Martínez-Moro et al., 2022; Zhu et al., 2025). For instance, in bovine embryos, factors such as HBE1, ITGA1, PAPPA, AKAP12, ITGA5, PSMB8, and SFRP4 have been identified (Martínez-Moro et al., 2022). Similarly, in human 8-cell embryos, decreased expression of factors involved in cell division—namely SMCO-1, ZNF271P, ZNF679, ASF1b, BEX3, DPPA2, and ORC4—has been associated with impaired blastocyst formation (Balboula et al., 2010). Similarly, in human 8-cell embryos, decreased expression of factors involved in cell division—namely SMCO-1, ZNF271P, ZNF679, ASF1b, BEX3, DPPA2, and ORC4—has been associated with impaired blastocyst formation (Balboula et al., 2010). Some of these newly discovered factors are currently being evaluated for their potential in assessing oocyte and fertilized egg quality.

Despite these technological advances, many aspects of early embryonic development remain elusive. In such cases, screening strategies employing inhibitor libraries that target various physiologically active pathways remain highly effective for identifying novel regulatory factors. For example, if among a panel of inhibitors only a low-molecular-weight inhibitor targeting enzyme “X” exhibits an effect on preimplantation development, it is reasonable to infer that enzyme “X” plays a role in the process. Thus, screening experiments using comprehensive inhibitor libraries may reveal previously unrecognized modulators of embryonic development and potentially lead to the development of novel molecular therapeutics (Kamata et al., 2019; Tokunaga et al., 2020). However, screening experiments to identify mammalian preimplantation embryonic regulatory factors using inhibitor libraries have been infrequently performed, in part due to the challenges of simultaneously processing large numbers of fertilized eggs derived from the same parents.

In this study, we developed a novel screening method by combining ultra-superovulation techniques (Takeo and Nakagata, 2015) with one-cell stage embryo cryopreservation technology to screen an inhibitor library for new regulators of preimplantation development. As a result, we identified the involvement of a p53 activator (PRIMA-1), cathepsin D, C-X-C motif chemokine receptor 2 (CXCR2), and potassium channels (apamin-sensitive K+ channels; SK2 and SK3) in embryonic development. These findings were further validated through CRISPR-Cas9 mediated knockout experiments. Collectively, our results suggest the existence of a novel group of regulatory factors in preimplantation development and highlight the potential for manipulating embryonic development through their modulation.

## 2 Materials and equipment

### 2.1 Animals

For the preparation of fertilized eggs, we used C57BL/6N male mice (8 weeks old) and female mice (4 weeks old) purchased from Japan SLC (Hamamatsu, Japan). All animals were maintained under specific pathogen-free conditions in individually ventilated cages (TECNIPLAST S. p.A., Buguggiate, Italy) on a 12-h light/12-h dark cycle at 22 °C ± 2 °C and 40%–60% relative humidity. They were provided with Labo MR Stock food (Nosan Corporation, Yokohama, Japan) *ad libitum*. All animal protocols were reviewed and approved by the Institutional Animal Care and Use Committee of Kanazawa Medical University and conducted in accordance with the university’s guidelines.

### 2.2 Preparation and cryopreservation of one-cell stage embryos

For all embryo culture experiments, one-cell stage C57BL/6N embryos were cryopreserved and subsequently thawed for use. The cryopreservation and thawing protocols have been described in our previous publication (Darwish et al., 2019). Briefly, to collect oocytes, 4-week-old C57BL/6N female mice were intraperitoneally injected with HyperOva (KYUDO CO., Ltd., Tosu, Japan) to induce ultra-superovulation. Forty-eight hours later, 7.5 IU of human chorionic gonadotropin (Sigma-Aldrich, St. Louis, MO, United States) was injected, and oocytes were harvested from the oviducts 16 h thereafter. Fertilization was performed in HTF medium (ARK Resources, Kumamoto, Japan) designed for *in vitro* fertilization, by incubating oocytes with sperm. Four hours after fertilization, excess sperm were removed, and the embryos were incubated in a 200 µL HTF drop supplemented with 20% fetal bovine serum for 10 min. Subsequently, the embryos were cryopreserved in liquid nitrogen using a freezing solution containing 1 M DMSO (ARK Resources) and DAP213 solution (ARK Resources). Thawing of the frozen embryos was rapidly carried out using 900 µL of 0.25 M sucrose solution, followed by two washes in KSOM medium (ARK Resources) before being used in subsequent experiments. To assess the effects of ultra-superovulation and freeze–thaw procedures, *in vivo* fertilized zygotes obtained after natural mating served as controls.

### 2.3 Preparation for the inhibitor library

We utilized two standardized inhibitor libraries, SCADS Inhibitor Kit II ver. 2.0 and SCADS Inhibitor Kit III ver. 1.6, which systematically compiles a broad range of chemical inhibitors commonly used in chemical biology research. A complete list of the inhibitors included in these kits is provided in Supplementary Table S1. The two SCADS inhibitor kits were obtained from the Molecular Profiling Committee of the Advanced Animal Model Support platform, Japan.

Each kit was supplied in a 96-well plate format, with 5 μL (10 nmol) of each inhibitor pre-dispensed per well. To prepare 100 μM stock solutions, 95 μL of 50% methanol was added to each well. These stock solutions were then diluted with KSOM medium, which was used for the subsequent embryo culture experiments.

### 2.4 Screening using the inhibitor library

For each embryo culture experiment, 20 thawed one-cell stage embryos were used per treatment group. Each inhibitor was added to KSOM medium at a final concentration of 1 μM, and embryos were cultured in this medium. In total, 96 experimental groups were prepared, including 95 inhibitor-treated groups and one control group. Each group was independently replicated three times, resulting in 288 embryo culture experiments.

The developmental rate (%) was calculated by dividing the number of developed embryos by the total number of embryos used in the experiment at the time of observation and then multiplying by 100. Mathematically, this is expressed as:

$$\text{Developmetal rate }\left(\%\right)=\left(\frac{N_{developed}}{N_{total}}\right)\times 100$$

where *N*_developed_ is the number of developed embryos, and *N*_total_ is the total number of embryos used in the experiment.

### 2.5 Analysis of RNA expression levels across human and mouse

To analyze expression levels of Cxcr2 and Ctsd genes across human and mouse embryonic development stages, we re-analyzed the GSE44183 (Xue et al., 2013) single-cell RNA-seq dataset. FASTQ files were downloaded from the Sequence Read Archive using SRA Toolkit and quantified using Salmon v1.10.0 (Patro et al., 2017) NCBIBioinformatics Answers with bias correction options (--gcBias, --seqBias, --validateMappings) against a combined human (GRCh38) and mouse (GRCm39) transcriptome reference from Ensembl release 114. Transcript-level abundances were imported and summarized to gene-level using tximport v1.30.0 (Soneson et al., 2015) GSE44183 (GEO), with transcript-to-gene mapping obtained via biomaRt. Differential expression analysis between species and developmental stages was performed using DESeq2 v1.42.0 (Love et al., 2014) Salmon: Accurate, versatile and ultrafast quantification from RNA-Seq data using lightweight-alignment | Request PDF with default parameters. Expression levels were visualized as log2(TPM+1) values and statistical significance was assessed at an adjusted p-value threshold of 0.05.

### 2.6 Immunofluorescence staining

Immunofluorescence staining of mouse embryos was based on the method of Sauvegarde et al. (Sauvegarde et al., 2016). For CTSD detection, an anti-CTSD rabbit polyclonal IgG antibody (1:200, 55021-1-AP, Proteintech group Inc., IL, United States), an anti-CXCR2 (1:200, 19538-1-AP, Proteintech) for CXCR2 detection, and as a negative control, a rabbit IgG isotype control antibody (98136-1-RR, Proteintech) was used. Anti-rabbit IgG recombinant VHH CoraLite® Plus 488 (1:500, srb2GCL488-1, Proteintech) was used as the secondary antibody. After the secondary antibody reaction, embryos were washed and then embryos were mounted on a 35-mm glass-bottom dish (3960–035, AGC TECHNO GLASS Co. Ltd., Shizuoka, Japan) and examined using an All-in-one fluorescence microscope (BZ-X1000, KEYENCE, Osaka, Japan).

### 2.7 Genome editing of one-cell stage embryos

Genome editing experiments were performed as previously described (Darwish et al., 2019; Nishizono et al., 2020). Briefly, CRISPR-Cas9 ribonucleoprotein (RNP) complexes were prepared by combining two guide RNAs (each consisting of 100 ng/μL crRNA and 200 ng/μL tracrRNA) with 100 ng/μL Cas9 protein in Nuclease-Free Duplex buffer (Integrated DNA Technologies, Inc., Coralville, IA, United States). Approximately 100 one-cell stage embryos were placed in the solution, and electroporation was conducted using a NEPA21 electroporator (Nepa Gene Co., Ltd., Ichikawa, Japan) to introduce the RNP complexes into the embryos. All reagents, including Cas9 protein, RNA oligonucleotides, and buffer, were purchased from Integrated DNA Technologies.

The sequences of the crRNAs used and the PCR primers for genotyping knockout alleles are listed in Supplementary Table S2. Because electroporation itself can cause physical damage to embryos (Miyagoshi et al., 2025), control embryos were electroporated in buffer with Alt-R™ Cas9 Negative Control crRNA (1072544, Integrated DNA Technologies), tracrRNA and Cas9 protein. Genome editing experiments for each target gene were independently repeated seven times.

### 2.8 Statistical analysis

All numerical data are presented as mean ± standard deviation (SD). Statistical analyses for each experiment were performed using JMP® Pro 18.0.2 (JMP Statistical Discovery LLC, Cary, NC, United States). Heatmap visualization and clustering analysis of the inhibitor library screening data were conducted using Python 3 with the Seaborn library.

## 3 Results

### 3.1 Optimization of screening conditions for the inhibitor library

**FIGURE 1:**
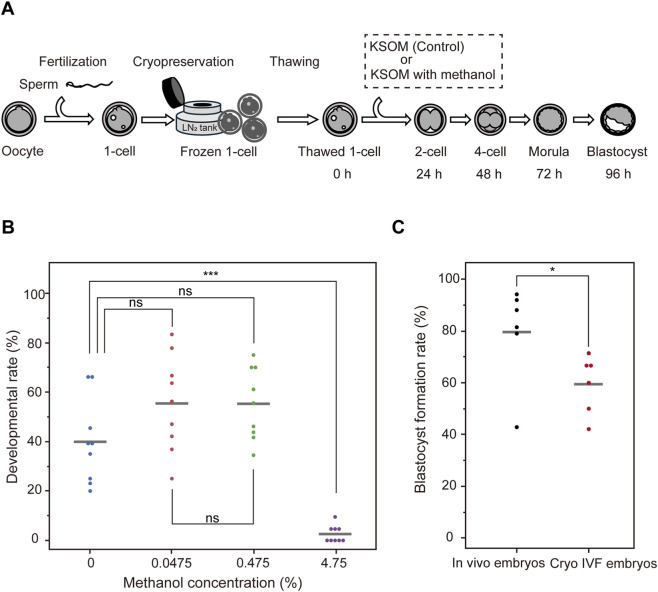
Optimization of methanol concentration for inhibitor library screening using mouse embryos. **(A)** Schematic workflow for testing methanol toxicity in mouse embryos. **(B)** Developmental rates of mouse embryos cultured for 96 h in KSOM medium containing methanol at various concentrations: 0% (control), 4.75% (standard final concentration from the stock solution), 0.475%, and 0.0475%. Embryo development was severely impaired at 4.75% methanol, whereas no significant differences were observed between the control and lower concentrations (0.475% and 0.0475%). Statistical analysis was performed using the Tukey-Kramer test (n = 3). **(C)** Blastocyst formation rates in embryos fertilized *in vivo* after natural mating (*In vivo* embryos) versus embryos generated by *in vitro* fertilization and subsequently cryopreserved (Cryo IVF embryos). Statistical analysis was performed using the t-test (n = 6). Data are presented as mean ± SD. ***p < 0.0001; *p < 0.05; n. s., not significant.

Prior to conducting the inhibitor library screening, we first evaluated the effects of the solvent used in the library on mouse fertilized eggs. The inhibitor library used in this study was prepared in 50% methanol, resulting in a stock solution concentration of 47.5%. To assess methanol tolerance, we tested its impact on embryo development at various final concentrations (Figure 1A). Under the standard usage condition, which yields a final methanol concentration of 4.75%, embryo development rates at 96 h after culture initiation were severely impaired, with a mean of 2.60% ± 3.43% (Figure 1B), indicating near-complete developmental arrest. This data suggests that mouse embryos are more sensitive to methanol toxicity compared to other cell types.

In contrast, methanol concentrations of 0.475% and 0.0475% (corresponding to 1/10 and 1/100 of the standard usage, respectively) did not show statistically significant differences in developmental rates compared to embryos cultured without methanol: 0.475% group: p = 0.1481; 0.0475% group: p = 0.1439, Tukey-Kramer test. Furthermore, no significant difference was observed between the 0.475% and 0.0475% methanol groups (p = 1.0000). The corresponding inhibitor concentrations at these dilutions were 1 μM (0.475% methanol) and 100 nM (0.0475% methanol), respectively. Based on these findings, we conducted all subsequent screening experiments using 1 μM inhibitors in 0.475% methanol diluted in KSOM medium. Furthermore, to evaluate whether ultra-superovulation and freeze–thaw procedures affect inhibitor efficacy, we compared *in vivo* fertilized embryos (*in vivo* embryos) with IVF-derived embryos obtained after ultra-superovulation and freeze–thawing (Cryo IVF embryos) under DMSO (1A) treatment (Figure 1C, n = 6). This analysis demonstrated a significant pre-stress in the Cryo IVF embryos (p < 0.05). Accordingly, subsequent analyses should be interpreted in the context of baseline pre-stress in the ultra-superovulation and freeze–thaw group.

### 3.2 Identification of novel regulators of embryonic development via inhibitor library screening

**FIGURE 2:**
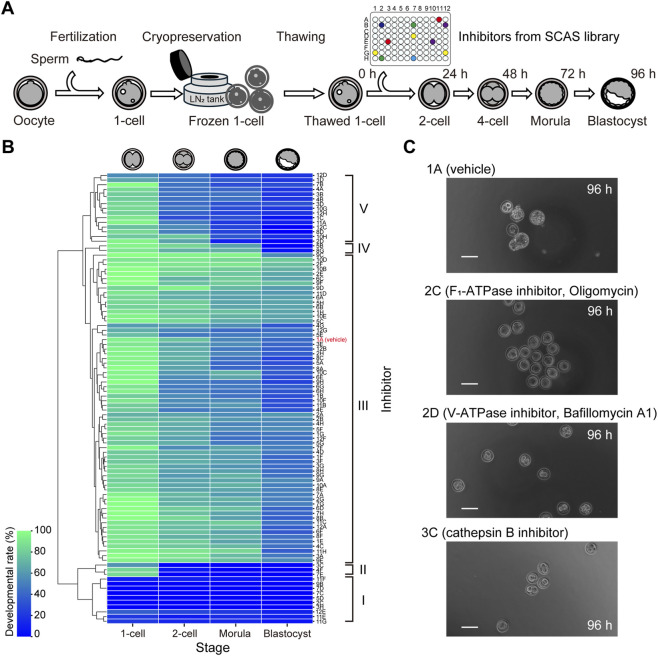
Screening of inhibitor libraries identified stage-specific regulators of early mouse embryonic development. **(A)** Schematic overview of the screening strategy using ultra-superovulation, *in vitro* fertilization, and one-cell embryo cryopreservation to ensure genetic uniformity across experimental groups. **(B)** Heatmap showing developmental outcomes of embryos cultured with 95 different inhibitors. Compounds were grouped by hierarchical clustering based on their stage-specific effects: Group I (lethal at one-cell stage), Group II (block at 1-cell to 2-cell), Group III (no effect), Group IV (block at blastocyst formation), and Group V (block at 4-cell to morula transition). Color scale represents the percentage of embryos reaching each developmental stage at 96 h, with blue indicating low and green indicating high developmental rates. **(C)** Representative developmental outcomes for selected known inhibitors. Scale bar = 100 μm.

Using the optimized inhibitor and solvent concentrations established in preliminary experiments, we screened for compounds that affect early embryonic development. To minimize genetic variability from parental origin, we employed a strategy that combined ultra-superovulation and *in vitro* fertilization (IVF) to generate large cohorts of fertilized eggs from the same parents. These one-cell stage embryos were cryopreserved, allowing us to conduct high-throughput screening using embryos with a uniform genetic background (Figure 2A).

Through this screening, we identified 16 compounds that significantly impaired embryo development, including five known regulators (Figure 2B). Hierarchical clustering analysis revealed that the inhibitors could be categorized into five groups based on their effects on developmental progression: Group I–inhibitors that caused severe toxicity at the one-cell stage; Group II–those that blocked development from the one-cell to two-cell stage; Group V–those that interfered with the transition from the 4-cell to morula stage; Group IV–those that impaired blastocyst formation; and Group III–those with minimal or no effect on development. Among the 95 inhibitors tested, the majority (68.42%) fell into Group III, followed by Group V (15.79%). In contrast, Group II (3.16%) and Group IV (2.11%) were relatively rare.

Of the five known factors identified, four were involved in energy metabolism: two ATPases and two mitochondrial complex inhibitors. Notably, inhibitors targeting F1-ATPase or mitochondrial complex I/III exerted strong deleterious effects at the one-cell stage, while V-ATPase inhibition specifically disrupted morula formation (Figure 2C). The remaining known factor, cathepsin B, markedly inhibited the transition from the one-cell to two-cell stage. In addition to these, we discovered 11 previously uncharacterized compounds that significantly impaired development at distinct stages, including inhibitors of p53 activator (PRIMA-1), cathepsin D, CXCR2, and potassium channels (Table 1).

**TABLE 1:** List of inhibitors exhibiting strong inhibitory effects on embryonic development of fertilized eggs.

Inhibitor		Embryonic developmental stage inhibited	Discovery status
ID	Name		
2C	F_1_-ATPase inhibitor, Oligomycin	1-cell stage	Known (Kane et al., J Reprod Fertil. 1977)
3H	CXCR2 inhibitor	1-cell stage	Novel
5D	K ionophore, Valinomycin	1-cell stage	Novel
7C	Mitochondrial complex I inhibitor	1-cell stage	Known (Kane et al., J Reprod Fertil. 1977)
7D	Mitochondrial complex III inhibitor	1-cell stage	Known (Kane et al., J Reprod Fertil. 1977)
9B	HIF inhibitor	1-cell stage	Novel
3C	Cathepsin B inhibitor	1-cll to 2-cell stage	Known (Afonso et al., Development 1997)
4F	Na ionophore	1-cll to 2-cell stage	Novel
7E	CRM1 inhibitor, Leptomycin B	1-cll to 2-cell stage	Novel
1C	p53 activator inhibitor, PRIMA-1	4-cll to morula stage	Novel
2D	V-ATPase inhibitor, Bafillomycin A1	4-cll to morula stage	Known (Inoue et al., Biochim Biophys Acta. 1999)
3D	Cathepsin D inhibitor	4-cll to morula stage	Novel
8D	Glycosylation inhibitor	4-cll to morula stage	Novel
12C	RNA polymerase inhibitor	4-cll to morula stage	Novel
5B	K channel inhibitor, Dequalinium	Morula to blastocyst stage	Novel
8G	Guanylate cyclase inhibitor, LY83583	Morula to blastocyst stage	Novel

**FIGURE 3:**
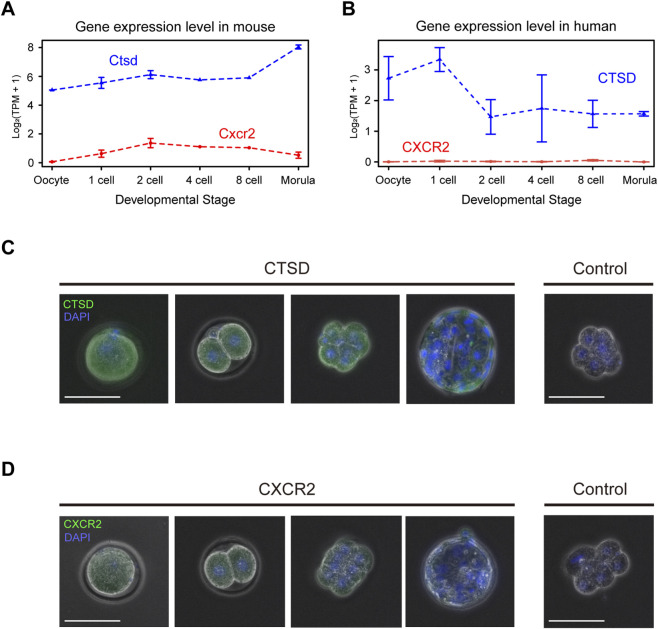
Cathepsin D (*Ctsd*) and *Cxcr2* genes are expressed in mouse embryo. **(A)** Expression level of Ctsd, and Cxcr2 by developmental stage in mice. The RNA-seq data from oocyte to morula stage mouse embryo in public database is reanalyzed. Each line indicates the expression level calculated by log2(TPM+1) values. **(B)** Expression level of CTSD, and CXCR2 by developmental stage in human. The RNA-seq data from oocyte to morula stage mouse embryo in public database is reanalyzed. Each line indicates the expression level calculated by log2(TPM+1) values. **(C)** Immunofluorescence staining of Ctsd protein (CTSD) from MII stage oocyte to blastocyst stage embryo. Green indicates CTSD, and blue indicates DAPI The right panel shows the result using a negative control antibody instead of the anti-CTSD antibody. Size bars: 100 µm. **(D)** Immunofluorescence staining of Cxcr2 protein (CXCR2) from MII stage oocyte to blastocyst stage embryo. Green indicates CXCR2, and blue indicates DAPI The right panel shows the result using a negative control antibody instead of the anti-CXCR2 antibody. Size bars: 100 µm.

Among them, cathepsin D inhibition interfered with progression from the 4-cell to morula stage, while CXCR2 inhibition affected the one-cell stage. RNA-seq analysis showed that Cathepsin D (*Ctsd*) transcripts were detectable from the oocyte stage through the morula stage, whereas *Cxcr2* transcripts, although near the detection limit, were present from the two-cell stage through the morula stage (Figure 3A). In human embryos, similar analyses indicated that CTSD was detected but with a distinct temporal profile, while CXCR2 transcripts were not detected above background (Figure 3B). Protein-level assessment by immunofluorescence (IF) corroborated these trends. CTSD signal was observed from the oocyte stage through the blastocyst stage, broadly consistent with the transcript data (Figure 3C). CXCR2 IF signal was very faint but reproducibly detectable from the two-cell stage through the morula stage, and it was not detected in blastocysts (Figure 3D). Based on these findings, we selected cathepsin D and CXCR2 for further validation using gene knockout experiments.

### 3.3 CRISPR-Cas9 mediated knockout of cathepsin D and CXCR2 genes in mouse embryos

**FIGURE 4:**
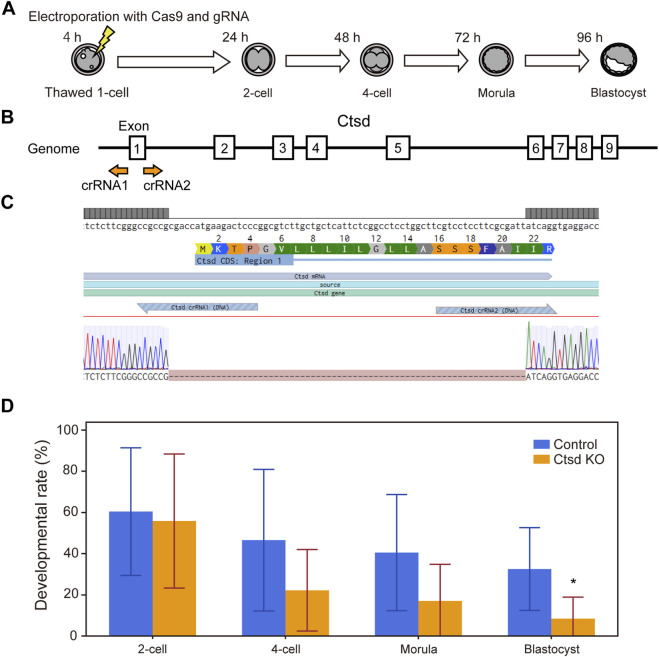
CRISPR-Cas9 mediated knockout of *Ctsd* genes in mouse embryos impairs preimplantation development. **(A)** Experimental workflow for CRISPR-Cas9 mediated gene knockout in one-cell stage embryos via electroporation. **(B)** Schematic of the *Ctsd* gene structure. Guide RNAs were designed to target the start codon in exon 1, which is shared across all splicing isoforms. **(C)** DNA sequences of the wild type (upper) and the knockout embryo (lower). Dashes (−) indicate nucleotides missing from the original target sequence. **(D)** Developmental outcomes of *Ctsd* knockout embryos. Data are presented as mean ± SD from three independent experiments. Statistical analysis was performed using the t-test. *p < 0.05.

**FIGURE 5:**
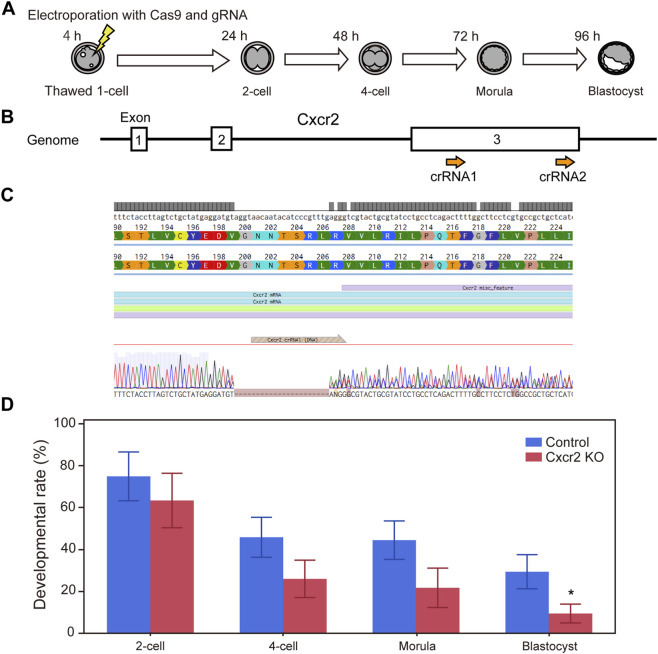
CRISPR-Cas9 mediated knockout of *Cxcr2* genes in mouse embryos impairs preimplantation development. **(A)** Experimental workflow for CRISPR-Cas9 mediated gene knockout in one-cell stage embryos via electroporation. **(B)** Schematic of the *Cxcr2* gene structure. Guide RNAs were designed to target exon 3, which contains the entire coding sequence. **(C)** DNA sequences of the wild type (upper) and the knockout embryo (lower). Dashes (−) indicate nucleotides missing from the original target sequence. **(D)** Developmental progression of *Cxcr2* knockout embryos. Data are presented as mean ± SD from three independent experiments. Statistical analysis was performed using the t-test. *p < 0.05.

To verify that the developmental arrest observed with specific inhibitors was attributable to inhibition of their intended targets, we performed loss-of-function experiments using the CRISPR-Cas9 system. Cas9 protein and guide RNAs (crRNA and tracrRNA) targeting either *Ctsd* (Figure 4) or *Cxcr2* (Figure 5) were delivered into one-cell-stage embryos by electroporation.

For *Ctsd*, because the gene is long and contains multiple exons and splice variants, we designed guides to the start codon in exon 1, which is conserved across all isoforms (Figure 4B). After electroporation, zygotes were collected for PCR and Sanger sequencing, which confirmed genomic deletions at the predicted cut sites (Figure 4C, genome editing efficiency = 100%). Developmental outcomes for these embryos versus those electroporated with a negative-control gRNA are shown in Figure 4D. *Ctsd* knockout embryos exhibited a decline in developmental rate from the 4-cell stage, with a significant reduction at the blastocyst stage (n = 7, p < 0.05). This phenotype was slightly delayed relative to the morula-stage arrest seen with pharmacologic inhibition (Figure 2B).

For *Cxcr2*, because the entire coding sequence resides within exon 3, we targeted this exon to achieve functional disruption (Figure 5B). Sanger sequencing indicated that double-strand breaks occurred within the region targeted by crRNA1 (of the two crRNAs tested), resulting in a partial deletion predicted to introduce a premature stop codon (Figure 5C, genome editing efficiency = 75%). *Cxcr2* knockout embryos showed a significant reduction in blastocyst formation (n = 7, p < 0.05).

Although the onset of developmental failure in both knockout models was slightly later than with small-molecule inhibition, these results collectively support essential roles for *Ctsd* and *Cxcr2* during early embryonic development.

## 4 Discussion

In this study, we demonstrated that our developed screening system enables efficient identification of novel factors involved in early embryonic development. We discovered 11 previously unreported regulators, including cathepsin D and CXCR2 (Figure 2; Table 1) using this inhibitor library-based approach. The developmental arrest induced by these compounds was further validated by CRISPR-Cas9 mediated gene knockout experiments, confirming their functional relevance (Figures 4, 5).

It is currently reported that one in six people will experience infertility during their lifetime (Cox et al., 2022). To address unexplained infertility and advance assisted reproductive technologies (ART), it is essential to uncover novel molecular players involved in fertilization, early embryonic development, and implantation (Sang et al., 2023). We identified critical roles for p53 activators, cathepsin D, CXCR2, and potassium channels (apamin-sensitive K+ channels; SK2 and SK3) in early embryonic development by applying our screening platform (Figure 2; Table 1). Interestingly, the potential involvement of the p53 pathway in early development has been previously suggested by proteomics studies in bovine embryos (Deutsch et al., 2014), and our findings provide direct functional evidence supporting this study. Moreover, we showed that different types of ATPases, already known to be important for development, arrest embryonic development at distinct stages (Figure 2C), offering insight into stage-specific energy requirements in fertilized eggs.

Cathepsins are proteases that cleave peptide bonds at serine, cysteine, or aspartic acid residues and play essential roles in digestion, immunity, adipogenesis, hormone release, peptide processing, apoptosis, and autophagy (Patel et al., 2018). To date, 15 cathepsin family members have been identified, but only cathepsin B and L have been implicated in embryonic development (Afonso et al., 1997). Cathepsin B, a cysteine protease, is essential for blastocyst formation, which was consistent with our results (Figure 2C). Interestingly, previous studies in bovine (Balboula et al., 2010) and ovine embryos (Pezhman et al., 2017) reported that inhibition of cathepsin B activity enhanced embryonic development and cryotolerance. The discrepancy with our results is likely due to differences in inhibitor concentration, suggesting that cathepsin B activity may be required in embryo within an optimal concentration range.

Cathepsin D, a lysosomal aspartyl protease, is known to be expressed in embryos (Li et al., 2020), yet its functional significance during embryonic development had not been clarified. While cathepsin D has been also associated with disorders such as neuronal ceroid lipofuscinosis, its intracellular roles remain poorly understood (Patel et al., 2018). In our study, Cathepsin D inhibition or *Ctsd* knockout impaired development from the 4-cell to morula stage (Table 1), suggesting a role in compaction or cell division during this window. The delayed phenotype observed in genome-edited embryos compared to inhibitor-treated ones may indicate that *Ctsd* mRNA is maternally loaded and persists through the one-cell stage. Indeed, *Ctsd* transcripts have been shown to accumulate at the one-cell stage in bovine embryos (Li et al., 2020). In avian species, cathepsin D is involved in yolk protein processing (Sheng et al., 2013), suggesting that similar mechanisms may exist in mammalian oocytes and preimplantation embryos. Although cathepsin L has also been reported to function in fertilized eggs (Ezz et al., 2024), it was not included in library we used this study. In contrast, cathepsin G was screened but showed no detectable effect on embryonic development (Figure 2B), suggesting the presence of stage-specific lysosomal protease repertoires in early preimplantation embryos.

CXCR2 belongs to the family of 20 identified chemokine receptors, and its most well-known ligands include CXCL8 (interleukin-8, IL-8) and CXCL1 (growth-regulated oncogene-α, GRO-α) (Korbecki et al., 2022). It is a seven transmembrane G protein–coupled receptor that not only activates downstream G protein signaling but also interacts with multiple intracellular partners, playing key roles in cancer and aging (Acosta et al., 2008). Interestingly, in the context of fertilized eggs, CXCR2 has been implicated in ameliorating the phenotype of aged oocytes upon inhibition (Kawagoe et al., 2020). However, its function in non-aged oocytes remained unknown. Our data suggests that CXCR2 signaling is critical for maintaining embryo viability during early cleavage (Figures 2-5). Recently, CXCR12, a ligand for CXCR4, has been proposed as an “embryokine” secreted from the oviduct that influences early embryonic development (Maldonado et al., 2025). Our findings raise the possibility that CXCR2 may also have its own oviduct-derived ligand functioning as an embryokine. However, the mechanism by which CXCR2 contributes to embryonic development, together with the identity and source of its ligands, remains unclear and requires further investigation. Notably, we observed cross-species differences between mice and humans in the expression of CTSD and CXCR2 in fertilized embryos (Figures 3A,B). These findings caution against direct extrapolation of our screening results to humans and suggest species-specific requirements for factors essential to early embryonic development.

A key limitation is that the platform relies on ultra-superovulation and freeze–thawing, which can impose baseline pre-stress on embryos; accordingly, inhibitor responses reported here should be interpreted in light of this potential confound and, where feasible, corroborated in vivo fertilized, non-cryopreserved embryos. In the discovery phase we screened 95 inhibitors at a single, relatively high concentration (1 μM), with uniform vehicle exposure, and performed confirmatory re-tests; in total, more than 6,000 embryos were analyzed. This design prioritized sensitivity to potential effects and comparability across conditions within the practical throughput constraints of embryo-based assays. We acknowledge that the lack of full dose–response curves limit precise potency estimation and may overcall weak or nonspecific effects, particularly given the baseline pre-stress associated with ultra-superovulation and freeze–thaw procedures. As next steps, we will conduct multi-point dose-response analyses, siRNA-based knockdown and validate prioritized hits in non-cryopreserved, *in vivo* fertilized embryos.

In summary, our screening system using cryopreserved one-cell embryos and chemical libraries successfully identified novel regulators of embryonic development, particularly stage-specific functions of cathepsin D and CXCR2. Expanding this approach to broader inhibitor libraries may uncover additional developmental regulators. Furthermore, applying this system to species other than mice holds potential for identifying species-specific embryonic factors. As future directions, this system may be applied in parallel to two distinct areas: screening traditional herbal medicine libraries for bioactive compounds that elude conventional genomic or proteomic analysis and identifying novel agents that could contribute to the development of emergency contraceptives.

## Data Availability

The original contributions presented in the study are included in the article/Supplementary Material, further inquiries can be directed to the corresponding author.
