# Supplementary material for: Screening for novel factors involved in mouse early embryonic development using inhibitor libraries
Source: Front Cell Dev Biol. 2025 Oct 17;13:1643551. doi: 10.3389/fcell.2025.1643551 (PMC12575340; doi:10.3389/fcell.2025.1643551)
Supplement: Supplementary file 1 [file Table1.docx]

Supplementary Material

Screening for Novel Factors Involved in Mammalian Early Embryonic Development Using Inhibitor Libraries

Hirofumi Nishizono^1^*

^1^ Research Support Center, Medical Research Institute, Kanazawa Medical University, Uchinada, Japan

* Correspondence:

Hirofumi Nishizono

hirofumi@kanazawa-med.ac.jp

# Supplementary Tables

## Supplementary Table S1. List of inhibitors used in this study.

| Well | | Category | Compound | Distributor | Product No. |
| --- | --- | --- | --- | --- | --- |
| 1- | A | blank | none (DMSO) |  |  |
| 1- | B | p53 | Pifithrin-a (cyclic) | Calbio　Chem | 508-43391 |
| 1- | C | p53 activator | PRIMA-1 | ALEXIS | 270-310-M001 |
| 1- | D | 5α-reductase | Finasteride | LKT | F3354 |
| 1- | E | aromatase | Aminoglutethimide | MPB | 153645 |
| 1- | F | aromatase | Formestane | LKT | F5769 |
| 1- | G | progesterone receptor | Mifepristone | TOCRIS | 576-77361 |
| 1- | H | acetyl-CoA carboxylase (ACC) | TOFA | Calbio　Chem | 613450 |
| 2- | A | aminopeptidase A | Amastatin | ALEXIS | 260-003-M001 |
| 2- | B | aminopeptidase M | Actinonin | ALEXIS | 260-128-M005 |
| 2- | C | F1-ATPase | Oligomycin | WAKO | 159-02181 |
| 2- | D | V-ATPase | Bafilomycin A1 | WAKO | 029-11643 |
| 2- | E | Bcl-2 | HA 14-1 | Calbio　Chem | 371971 |
| 2- | F | Bcl-XL | BH3I-1 | ALEXIS | 430-122-M005 |
| 2- | G | Burton's tyrosine kinase (BTK) | LFM-A13 | CAYMAN | 10010265 |
| 2- | H | Burton's tyrosine kinase (BTK) | Terreic acid | TOCRIS | 557-75851 |
| 3- | A | Calpain | E-64d | Calbio　Chem | 330005 |
| 3- | B | calpain, cathepsin B, L | ALLN | MPB | 598-00131 |
| 3- | C | Cathepsin B | CA-074 | PEPTIDE INSTITUTE, INC. | 337-43221 |
| 3- | D | Cathepsin D | Pepstatin A | PEPTIDE INSTITUTE, INC. | 4397-v |
| 3- | E | Cathepsin G | Z-GLF-CMK | SIGMA | C9984 |
| 3- | F | CCR2 | RS 102895 | TOCRIS | 2089 |
| 3- | G | CCR3 | SB 328437 | Calbio　Chem | 559406 |
| 3- | H | CXCR2 | SB 225002 | Calbio　Chem | 559405 |
| 4- | A | CXCR4 | AMD3100 octahydrochloride | SIGMA | A5602 |
| 4- | B | Cdc25 | NSC95397 | TOCRIS | 1547 |
| 4- | C | blank | none (DMSO) |  |  |
| 4- | D | Na channel | Amiloride | Calbio　Chem | 535-79211 |
| 4- | E | Na channel | Lidocaine | WAKO | 120-02691 |
| 4- | F | Na ionophore | Monensin | Calbio　Chem | 530-80371 |
| 4- | G | Na/K ATPase | Ouabain | ChromaDex, Inc. | ASB-00019365-010 |
| 4- | H | Na/K/Mg ATPase | Sanguinarine | ChromaDex, Inc. | ASB-00019050-010 |
| 5- | A | K channel | Glibenclamide | WAKO | 078-03881 |
| 5- | B | K channel | Dequalinium | SIGMA | D3768 |
| 5- | C | K channel opener | Diazoxide | SIGMA | D9035 |
| 5- | D | K ionophore | Valinomycin | WAKO | 228-01121 |
| 5- | E | K ionophore | Nigericin | LKT | N3225 |
| 5- | F | Ca channel | Diltiazem | WAKO | 047-20311 |
| 5- | G | Ca channel | Nifedipine | MPB | 591-08203 |
| 5- | H | Ca channel, MDR | Verapamil | WAKO | 222-00781 |
| 6- | A | MDR | PGP-4008 | ALEXIS | 270-290-M002 |
| 6- | B | BCRP | Fumitremorgin C | ALEXIS | 350-127-C250 |
| 6- | C | Ca ionophore | A23187 | WAKO | 019-20111 |
| 6- | D | Ca ionophore | Ionomycin | Stress Marq | SIH-228 |
| 6- | E | Ca-ATPase | Thapsigargin | WAKO | 209-17281 |
| 6- | F | Ca-ATPase | t-Butylhydroquinone (BHQ) | WAKO | 027-07212 |
| 6- | G | Cl channel | N-phenylanthranilic acid | WAKO | 164-01331 |
| 6- | H | Cl channel | DIDS | MPB | 592-03711 |
| 7- | A | Chk 1 | SB 218078 | Calbio　Chem | 559402 |
| 7- | B | Chk 1, 2 | Debromohymenialdisine (DBH) | ALEXIS | 350-290-C100 |
| 7- | C | mitochondrial complex I | Rotenone | MPB | 599-10811 |
| 7- | D | mitochondrial complex III | Antimycin A1 | MPB | 591-01221 |
| 7- | E | CRM1 | Leptomycin B* | Dr. Yoshida (RIKEN) |  |
| 7- | F | DAG kinase | R59022 | SIGMA | D5919 |
| 7- | G | DAG kinase | Dioctanoylglycol | TOCRIS | 538-43171 |
| 7- | H | DAG lipase | RHC80267 | MPB | 159011 |
| 8- | A | DAG acyltransferase (DGAT) | Xanthohumol | ALEXIS | 572-72961 |
| 8- | B | fatty acid synthase (FAS) | C75 | ALEXIS | 270-286-M001 |
| 8- | C | FAS | Cerulenin | WAKO | 031-18181 |
| 8- | D | glycosylation | Tunicamycin | WAKO | 202-08241 |
| 8- | E | glucosidase I, II | Deoxynojirimycin | Calbio　Chem | 260684 |
| 8- | F | a-mannosidase | Swainsonine | WAKO | 198-10281 |
| 8- | G | guanylate cyclase | LY 83583 | WAKO | 128-04691 |
| 8- | H | guanylate cyclase | ODQ | WAKO | 153-01981 |
| 9- | A | HAT | Anacardic acid | ALEXIS | 270-381-M005 |
| 9- | B | HIF | Chetomin | BioViotica | BVT-0161-M001 |
| 9- | C | HIF-1a hydroxylase | Dimethyloxalylglycine | CAYMAN | 71210 |
| 9- | D | kinesin Eg5 | HR22C16 | Calbio　Chem | 270-373-M001 |
| 9- | E | kinesin Eg5 | Monastrol | Calbio　Chem | 475879 |
| 9- | F | lipoxygenase | Nordihydroguaiaretic acid (NDGA) | MPB | 592-08451 |
| 9- | G | 12, 15-lipoxygenase | ETYA | CAYMAN | 90120 |
| 9- | H | 12-lipoxygenase | Baicalein | WAKO | 027-07751 |
| 10- | A | Mdm2 | Nutlin-3 | CAYMAN | 10004372 |
| 10- | B | Mdm2 | MDM2 inhibitor | Calbio　Chem | 444145 |
| 10- | C | monoamine oxidase | Phenelzine | USP | 1517006 |
| 10- | D | monoamine oxdase B | Deprenyl | MPB | 151469 |
| 10- | E | mitochondrial permeability transition pore (MPTP) | Decylubiquinone | MPB | 195041 |
| 10- | F | MPTP | Ro 5-4864 | SIGMA | C5174 |
| 10- | G | MPTP opener | Lonidamine | SIGMA | L4900 |
| 10- | H | myosin light chain kinase | ML-7 | Calbio　Chem | 475880 |
| 11- | A | O6-methylguanine-DNA methyltransferase (MGMT) | Benzylguanine | ALEXIS | 480-019-M010 |
| 11- | B | ornithine decarboxylase (ODC) | DFMO | Calbio　Chem | 507-28081 |
| 11- | C | PKG | KT 5823 | WAKO | 118-00594 |
| 11- | D | PKG | Rp-8-CPT-cGMPS | ALEXIS | 480-087-M001 |
| 11- | E | PPAR-a | MK 886 | WAKO | 130-13001 |
| 11- | F | PPAR-a activator | Clofibrate | WAKO | 039-10603 |
| 11- | G | PPAR-g | BADGE | TOCRIS | 1326 |
| 11- | H | PPAR-g activator | Troglitazone | Calbio　Chem | 571-71691 |
| 12- | A | reverse transcriptase | AZT | WAKO | 015-14704 |
| 12- | B | reverse transcriptase | Nalidixic acid | SIGMA | N4382 |
| 12- | C | RNA polymerase | a-Amanitin | Calbio　Chem | 129741 |
| 12- | D | telomerase | MST-312 | Calbio　Chem | 581011 |
| 12- | E | telomerase | b-Rubromycin | ALEXIS | BVT-0251-M001 |
| 12- | F | TGF-b receptor | SB 431542 | TOCRIS | 584-77601 |
| 12- | G | spermidine/spermine N1-acetyltransferase (SSAT) activator | N1,N12-Diethylspermine (BESpm) | MPB | 592-28471 |
| 12- | H | sphingosine N-acyltransferase | Fumonisin B1 | WAKO | 62580 |

## Supplementary Table S2. The crRNA and PCR primers used in the study.

| Ctsd crRNA1 | 5’-gggagtcttcatggtcgcggcgg-3’ |
| --- | --- |
| Ctsd crRNA2 | 5’-cgtcctccttcgcgattatcagg-3’ |
| Ctsd genotyping F primer | 5’-tgagggccaaacaagcgg-3’ |
| Ctsd genotyping R primer | 5’-catctccggacccagagc-3’ |
| Cxcr2 crRNA1 | 5’-aacaatacatcccgtttgaggg-3’ |
| Cxcr2 crRNA2 | 5’-cgaagatgacccgcatggcccgg-3’ |
| Cxcr2 genotyping F primer | 5’-gtcaagtttgtgtgcatagcca-3’ |
| Cxcr2 genotyping R primer | 5’-ggccttgtcaatgtcatcgc-3’ |
